# Is there any role of intravenous iron for the treatment of anemia in cancer?

**DOI:** 10.1186/s12885-016-2686-2

**Published:** 2016-08-20

**Authors:** Cengiz Gemici, Ozlem Yetmen, Gokhan Yaprak, Sevgi Ozden, Huseyin Tepetam, Hazan Ozyurt, Alpaslan Mayadagli

**Affiliations:** 1 Department of Oncology, Dr. Lutfi Kirdar Kartal Education and Research Hospital, Cevizli, Istanbul, Turkey; 2 Department of Oncology, Bezmialem Vakif University, Istanbul, Turkey

**Keywords:** Anemia, Solid tumor, Intravenous iron

## Abstract

**Background:**

Anemia is a major cause of morbidity in patients with cancer resulting in poor physical performance, prognosis and therapy outcome. The aim of this study is to assess the efficacy of intravenous (iv) iron administration for the correction of anemia, for the prevention of exacerbation of anemia, for decreasing blood transfusion rates, and for the survival of cancer patients.

**Methods:**

Patients with different solid tumor diagnosis who received iv iron during their cancer treatment were evaluated retrospectively. Sixty-three patients with hemoglobin (Hgb) levels between ≥ 9 g/dL, and ≤ 10 g/dL, and no urgent need for red blood cell transfusion were included in this retrospective analysis. The aim of cancer treatment was palliative for metastatic patients (36 out of 63), or adjuvant or curative for patients with localized disease (27 out of 63). All the patients received 100 mg of iron sucrose which was delivered intravenously in 100 mL of saline solution, infused within 30 min, 5 infusions every other day. Complete blood count, serum iron, and ferritin levels before and at every 1 to 3 months subsequently after iv iron administration were followed regularly.

**Results:**

Initial mean serum Hgb, serum ferritin and serum iron levels were 9.33 g/dL, 156 ng/mL, and 35.9 μg/dL respectively. Mean Hgb, ferritin, and iron levels 1 to 3 months, and 6 to 12 months after iv iron administration were 10.4 g/dL, 11.2 g/dL, 298.6 ng/mL, 296.7 ng/mL, and 71.6 μg/dL, 67.7 μg/dL respectively with a statistically significant increase in the levels (*p* < 0.001). Nineteen patients (30 %) however had further decrease in Hgb levels despite iv iron administration, and blood transfusion was necessary in 18 of these 19 patients (28.5 %). The 1-year overall survival rates differed in metastatic cancer patients depending on their response to iv iron; 61.1 % in responders versus 35.3 % in non-responders, (*p* = 0.005), furthermore response to iv iron correlated with tumor response to cancer treatment, and this relation was statistically significant, (*p* < 0.001).

**Conclusions:**

Iv iron administration in cancer patients undergoing active oncologic treatment is an effective and safe measure for correction of anemia, and prevention of worsening of anemia. Amelioration of anemia and increase in Hgb levels with iv iron administration in patients with disseminated cancer is associated with increased tumor response to oncologic treatment and overall survival. Response to iv iron may be both a prognostic and a predictive factor for response to cancer treatment and survival.

## Background

Anemia is an important and common problem in cancer patients. Besides affecting physical, functional, emotional well-being and quality of life, it has a negative impact on treatment outcome, prognosis and survival [1–4]. Bleeding, hemolysis, nutritional deficiencies, renal dysfunction with decreased erythropoietin synthesis, tumoral infiltration of bone marrow, myelosuppression from cancer treatment are among the common causes of anemia in cancer patients. Besides all the reasons mentioned above, probably the most important one for the development of cancer associated anemia is the presence of chronic inflammatory state and release of inflammatory cytokines related to the tumor itself [1–4]. These cytokines such as interleukin-6 result in erythroid progenitor cell suppression, impaired erythropoietin production, impaired iron utilization and decreased half-life of red blood cells [3, 5, 6]. Inflammatory cytokines play a role in iron metabolism through hepcidin synthesis, which is a liver produced protein, and has a primordial role in iron metabolism [5, 6]. Hepcidin modulates the release of iron from different cell sources, including enterocytes, macrophages, and hepatocytes to plasma. Through these effects, hepcidin controls iron absorption from the gut, the recycling of iron derived from senescent and damaged erythrocytes, and the release of iron from tissue stores [5, 6].

Anemia in cancer patients may be observed either by depletion of total body iron stores and low serum ferritin levels, which is called absolute iron deficiency (AID), or with normal or elevated total body iron stores and normal or elevated serum ferritin levels, which is called functional iron deficiency (FID) [3, 5]. Although oral iron prescription is a very common practice for anemia treatment in cancer patients, many of the patients still require blood transfusion despite adequate oral iron supplementation [7, 8]. Hepcidin mediated inhibition of gut absorption of iron explains why there is little or no response to oral iron supplementation [5, 6]. Intravenous iron usage in cancer patients is rare, and has been popularized with the approval of erythropoiesis-stimulating agents (ESAs) in 1997 in oncology, primarily to enhance the response to erythropoietin [9]. Accordingly, the first treatment guideline published for cancer associated anemia in 2002 was primarily for ESAs usage [9, 10]. After popularization of ESAs usage, iv iron has been used mostly as an adjunct to ESAs [5, 9, 11].

Intravenous administration of iron is more effective than its oral administration for correction of anemia especially in patients with FID, which results from failure to provide iron to the erythroblasts despite sufficient iron stores [5, 9]. One important reason for this failure is the trapped iron in the cells; neither dietary iron is released from the enterocytes in the small intestine, nor the stored iron is released from the cells of the reticulo-endothelial system (macrophages, liver) for erythropoiesis [9, 12]. The major mechanism behind FID is the cytokine-mediated increase in hepcidin levels which in turn reduces the normal function of ferroportin. Ferroportin is a cell surface transmembrane protein whose function is transfer of iron from the intracellular stores to transferrin, the transport protein of iron in the blood [9, 12]. Iv administration of iron may play a role in overcoming resistance to hepcidin related reduced iron availability to erythroblasts and ultimately correction of anemia in these patients. Iv iron can also overcome the problems of malabsorption of iron which is quite frequent in cancer patients, due to surgery, radiotherapy and chemotherapy.

Despite the better efficacy of the iv route of iron administration, the oral route is still the preferred way of administration among oncologists for the treatment of cancer associated anemia. There are serious concerns among oncologists regarding the iv iron utilization, like allergic reactions, accumulation of iron in tissues, lack of knowledge and lack of enough literature about the safety and efficacy of iv iron use in the treatment of cancer associated anemia.

We analyzed the role of iv iron administration on the outcome of patients with localized or metastatic cancer regarding anemia and survival parameters. The primary aim of the study was to find out if iv iron could prevent further exacerbation of anemia in patients undergoing active cancer treatment, increase the Hgb levels and decrease eventual and inevitable blood transfusion rates secondary to oncologic treatment.

## Methods

The medical records of patients with various malignancies who received iv iron during their cancer treatment were retrospectively evaluated. The study period was between January 2009 and January 2015. Only anemic patients with Hgb levels between ≥ 9 g/dL, and ≤ 10 g/dL, and who did not receive red blood cells transfusion before were included in this retrospective analysis. Among different reasons for intravenous administration of iron, the most common ones were either the refusal of blood transfusion by the patient, or the prevention of future blood transfusion secondary to worsening of anemia under oncologic treatment, or as an alternative to erthyropoietin use due to its prescription limitations. Sixtythree patients were identified, 36 had metastatic disease receiving palliative chemotherapy (CT), radiotherapy (RT) or both, while 27 had localized disease receiving either adjuvant or definitive treatment with CT, RT or concomitant chemoradiotherapy (CRT).

The most commonly administered chemotherapy combination during treatment of the patients was; Docetaxel + Cisplatin (±5FU) (23.8 %). The details of other chemotherapy schemas administered during the study are summarized in Table 1. CT was administered for a minimum of 3 cycles, either daily for oral chemotherapeutics, weekly, every 15 days, every 21 days or monthly cycles for iv administrations. CT was administered either alone for most of the metastatic patients or concomitantly with radiotherapy for patients with localized disease as curative or adjuvant treatment.

**Table 1:** Treatment characteristics

	Patients	Percent
Chemotherapy combinations		
Docetaxel + Cisplatin (±5FU)	15	23.8 %
FOLFOX	12	19 %
FEC	10	15.8 %
Paclitaxel + Carboplatin	7	11.1 %
Capecitabine	5	8 %
FUFA	2	3.1 %
Others	12	19.2 %
Radiotherapy areas		
Bony areas	15	23.8 %
Pelvic region	10	15.8 %
Thoracic region	7	11.1 %
Upper abdomen	4	6.3 %
Brain	1	1.5 %

*Abbreviations: 5FU* 5-Fluorouracil, *FOLFOX*, 5-Fluorouracil, Folinic Acid, Oxaliplatin, *FEC* 5-Fluorouracil, Epirubicin, Cyclophosphamide, *FUFA* 5-Fluorouracil, Folinic Acid

RT was administered to the upper abdomen, pelvic, thoracic region, or bony areas either alone or concurrently with CT in 37 out of 63 patients, either as part of adjuvant, curative or palliative treatment. Radiation dose was 30 Gy in 3 Gy fractions per day for palliative treatments, 46 to 60 Gy in 2 Gy fractions per day for adjuvant or curative treatments. Treatment details are summarized in Table 1.

Although the study was not randomized and not designed with a control group who did not receive iv iron, these patients have already generated their own controls with their Hgb levels before and after the administration of iv iron.

Only patients receiving treatments with CT, RT, or CRT were considered for the intervention of iv iron, while patients receiving no treatment for their cancer or followed regularly after any treatment were not included in this study.

Iv iron was administered as 100 mg iron sucrose (Venofer) in 100 mL of saline solution, within 30 min of infusion time, 5 infusions every other day. Five-hundred milligrams of iron sucrose was administered in total to all the patients while they were undergoing CT, RT or both.

The study was approved by the local ethics committee of the Dr. Lutfi Kirdar Kartal Education and Research Hospital. The patients were followed up regularly by physical examination and complete blood count, serum iron, and ferritin levels before, and at every 1 to 3 months subsequently after iv iron infusion.

Overall survival rates were calculated using the Kaplan-Meier method. Overall survival was measured from the date of intervention (iv iron administration), to the time of the last follow-up or date of death. Comparison of the survival curves between the groups was performed with the log-rank test. Repeated measures test, and chi-square test were used to determine the significance of response rate to iv iron administration between patients with metastatic and localized disease.

Univariate analysis was performed to evaluate the significance of age, gender, tumor type, and administration of iv iron in patients with metastatic cancers. A multivariate analysis was planned depending on the significance of the factors. Blood transfusion was performed in patients who did not respond to iv iron, thus it was not included in the multivariate analysis.

## Results

Sixty-three patients (34 female, median age 56 [24-81]) were identified. Demographics of the patients are summarized in Table 2.

**Table 2:** Patients characteristics

	Patients	%
Gender		
Female	34	54 %
Male	29	46 %
Median age	56 (range 24–81)	
Treatment type		
Adjuvant or curative	27	42.9 %
Metastatic	36	57.1 %
Cancer type		
Gastrointestinal cancers	20	31.7 %
Breast cancer	15	23.8 %
Lung cancers	11	17.5 %
Others	17	27 %
Mean levels	Before iv Iron	After iv Iron
Hemoglobin	9.33 g/dL	11.2 g/dL
Serum ferritin	156 ng/mL	296.7 ng/mL
Serum iron levels	35.9 μg/dL	67.7 μg/dL
Blood transfusion	18 (28.6 %)	

Most common tumor types were gastrointestinal cancers (31.7 %), followed by breast (23.8 %), lung (17.5 %), and other tumor types (27 %).

Before the administration of iv iron; mean Hgb level for the whole group was 9.33 ± 0.3 g/dL (range 9–10 g/dL), mean ferritin level was 156 ± 210 ng/mL (range 2–943 ng/mL), and mean serum iron level was 35.9 ± 23.1 μg/dL (range 9–107 μg/dL). One to three months after iv iron administration, the mean Hgb level was 10.4 ± 1.1 g/dL (range 8.6–13.4 g/dL), the mean ferritin level was 298.6 ± 283 ng/mL (range 6.4–1131 ng/mL), and mean iron level was 71.6 ± 41.4 μg/dL (range 10–276 μg/dL). Six to 12 months later, the mean Hgb level was 11,2 g/dL (range 8.2–15.1 g/dL), the mean ferritin level was 296.7 ng/mL (range 8–1600 ng/mL), and mean serum iron level was 67,7 μg/dL (range 10–235 μg/dL). The increase in Hgb, ferritin and iron levels after iv iron administration was statistically significant with a *p* value <0.001.

Increase in Hgb levels by iv iron administration was not temporary as in blood transfusion and were sustained throughout the study period. Iv iron administration increased Hgb levels both in metastatic patients, and also in patients with localized disease; 1.25 g/dL, and 2.5 g/dL successively within 6 to 12 months. Increase in Hgb levels was statistically significant for both groups with a *p* < 0.001. Treatment results are summarized in Tables 3, 4 and 5 for all the patients; patients with metastatic disease treated with palliative intent, and patients with localized disease treated with adjuvant or curative intent are reported separately.

**Table 3:** Treatment results in all patients

Baseline results		1–3 months results	6–12 months results	p:
Hemoglobin mean/range	9.33 g/dL (9–10 g/dL)	10.4 g/dL (8.6–13.4 g/dL)	11.2 g/dL (8.2–15.1 g/dL)	<0,001
Ferritin mean/range	156 ng/mL (2–943 ng/mL)	298.6 ng/mL (6,4–1131 ng/mL)	296.7 ng/mL (8–1600 ng/mL)	<0,001
Iron mean/range	35.9 μg/dL (9–107 μg/dL)	71.6 μg/dL (10–276 μg/dL)	67.7 μg/dL (10–235 μg/dL)	<0,001

**Table 4:** Treatment results in metastatic patients

Baseline results		1–3 months results	6–12 months results	P:
Hemoglobin mean/range	9.2 g/dL (9–9,7 g/dL)	9.9 g/dL (8.6–13.4 g/dL)	10.45 g/dL (8.2–13.5 g/dL)	<0,001
Ferritin mean/range	236 ng/mL (4–943 ng/mL)	410.3 ng/mL (45–1131 ng/mL)	425.9 ng/mL (10–1600 ng/mL)	0,003
Iron mean/range	42.6 μg/dL (13–107 μg/dL)	76.2 μg/dL (10–276 μg/dL)	61.5 μg/dL (10–178 μg/dL)	<0,001

**Table 5:** Treatment results in patients with localized disease

Baseline results		1–3 months results	6–12 months results	P:
Hemoglobin mean/range	9.5 g/dL (9–10 g/dL)	11 g/dL (9.3–12.6 g/dL)	12 g/dL (9.3–15.1 g/dL)	<0,001
Ferritin mean/range	49.3 ng/mL (2–296 ng/mL)	149.5 ng/mL (6.4–724 ng/mL)	124.4 ng/mL (8–796 ng/mL)	0,003
Iron mean/range	27 μg/dL (9–54 μg/dL)	65.4 μg/dL (23–103 μg/dL)	75.9 μg/dL (25–235 μg/dL)	<0,001

Nineteen out of 63 patients (30 %) did not respond to iv iron with further decrease in their Hgb levels within 3 months after iv iron administration, and 18 out of these 19 patients (28.5 %) received red blood cell transfusion due to worsening of the anemia and the appearance of anemia associated symptoms.

**Fig. 1:**
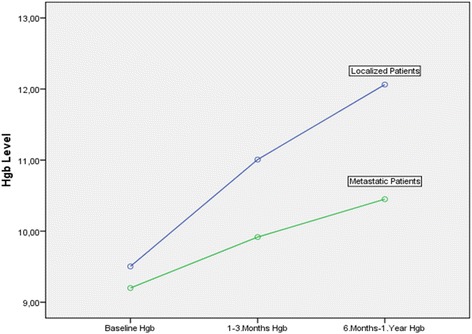
Estimated means of Hgb increase in patients with metastatic and localized disease after iv iron administration

Increase in Hgb levels after iv iron administration was more frequent in patients with localized disease treated either with adjuvant or curative intent (26 out of 27 patients) in comparison to metastatic patients (18 out of 36 patients) *p* < 0.001. Only 1 out of 27 patients with localized disease (3.7 %) presented with further decrease in Hgb levels despite iv iron administration, while 18 out of 36 patients with metastatic disease (50 %) presented with further decrease in Hgb levels, and the difference in Hgb decrease between metastatic and localized disease was statistically significant (*p* < 0.001). Estimated means of Hgb increase between patients presenting with metastatic and localized tumors is presented in Fig 1.

**Fig. 2:**
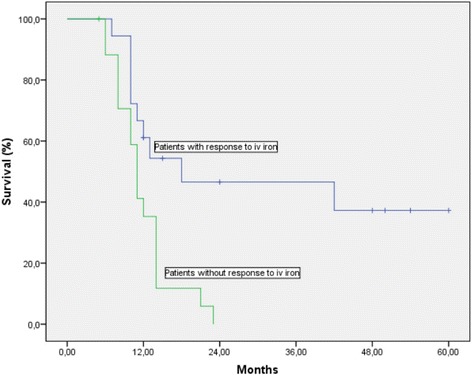
Survival curve of metastatic patients with and without increase in Hgb levels after iv iron administration

**Fig. 3:**
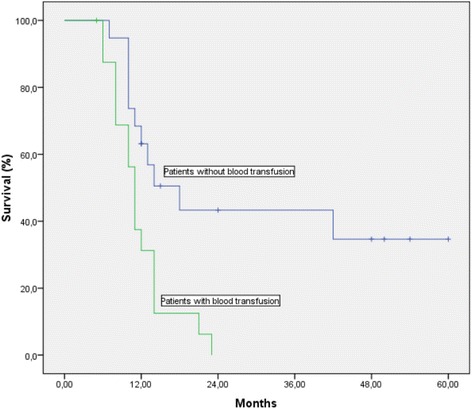
Survival curve of metastatic patients with and without red blood cell transfusion

Median follow up duration for all the patients was 43 months (range 6–60 months). Another important finding in this retrospective analysis was the survival difference in metastatic patients depending on their response to iv iron. There was statistically significant difference between 1-year survival rates in patients with and without increase in Hgb levels after iv iron administration (61.1 vs 35.3 %, *p* = 0.005) (Fig. 2). The 1-year survival difference was also statistically significant between metastatic patients who received red blood cell transfusion, and who did not during the study period (31.3 vs 63 %, *p* = 0.004), (Fig. 3). However the survival figures should be evaluated with caution since the group was not homogenous with respect to tumor, treatment and patient characteristics.

In metastatic patients, univariate analysis revealed that age, gender, tumor type were not statistically significant. The only factor that affected survival was the administration of iv iron (*p* < 0.001). Thus a multivariate analysis was not performed.

Another important finding in the study was the correlation of tumor response rates to cancer treatment with response to iv iron in patients presenting with metastatic disease. Thirteen out of 18 patients with metastatic disease who had increased Hgb levels with iv iron administration also showed response to their cancer treatment which was verified by RECIST criteria (response evaluation criteria in solid tumors). On the other hand, 17 out of 18 metastatic patients who did not respond to iv iron administration also did not respond to their cancer treatment either, *p* < 0,001. Table 6. summarizes the tumor response to cancer treatment with iv iron response in metastatic patients. We think that the response to iv iron is both predictive and prognostic for tumor response to cancer treatment and survival.

**Table 6:** Correlation of tumor response to cancer treatment with iv iron response in patients with metastatic disease

		Response to treatment		Total	p:
		Yes	No		
Response to iv iron	Yes	13	5	18	
	No	1	17	18	
Total		14	22	36	<0.001

## Discussion

Intravenous iron use in cancer related anemia has been popularized with the approval of erythropoiesis-stimulating agents in 1997 in oncology, and iv iron was shown to enhance the response to erythropoietin [5, 9, 11]. Since then ESAs and iv iron combination were commonly used for the treatment of cancer associated anemia. However after popularization of ESAs, certain toxicities associated with their usage, and increased mortality risk has been observed. Among them were increased thromboembolic risk, cardiovascular adverse events and stimulation of disease progression in tumor cells with the expression of erythropoietin receptors [13–15]. Although ESAs with or without iv iron reduced the need for red blood cell transfusions, 13 years after their approval in oncology, certain precautions have been suggested and their use was restricted due to the above mentioned adverse events [13, 16]. Unfortunately iv iron administration which was mostly used as an adjuvant to ESAs to treat cancer associated anemia has been abandoned with the prescription restrictions of ESAs. Red blood cell transfusions became popular again for the correction of cancer associated anemia. However blood transfusion is not devoid of toxicity and is as harmful as ESAs [17–19].

Iv iron on the other hand is a promising strategy and is reported to be an effective treatment for anemia of chronic diseases such as chronic renal failure, chronic kidney disease and cancer [20–22]. Iv iron may be an even more effective treatment alternative for anemia of chronic disease associated with inflammation like cancer, since intravenous administration may overcome resistance to iron absorption especially by erythroid cells and iron recycling which are all controlled by hepcidin.

Although iv iron has been demonstrated to be superior to oral iron in improvement of erythropoietic response to ESAs, there are limited studies of iv iron alone without ESAs in the treatment of cancer associated anemia [5, 9, 22–26]. Iv iron together with ESAs not only increase Hb levels higher than ESAs alone, but also in a shorter time interval than ESAs, besides these advantages, the addition of iv iron to ESAs decreases blood transfusion rates significantly compared to ESAs alone [5, 9, 24, 25].

The first study investigating iv iron alone in oncology practice was performed and published in 2007, and included women with cervical cancer treated with chemoradiotherapy [26]. The primary objective of this study was to prevent exacerbation of anemia and to reduce blood transfusion by iv iron. In this trial the transfusion rate dropped from 64 to 40 %. In 2010, another single-center, prospective, randomized study was published exploring the effect of iv iron administration on blood transfusion rates in anemic gynecologic cancer patients receiving platinum-based chemotherapy [22]. Again, this was a small study with 22 patients in each arm, but iv iron resulted in a significant Hgb increase of 0.9 g/dl and a significant reduction of the transfusion rate from 63.6 to 22.7 %. In both of these studies patients received iv iron regardless of their initial iron status.

We observed in our retrospective study that, iv iron provided a significant increase in Hgb levels in already anemic cancer patients undergoing oncologic treatment either with CT, RT or both. The increase in Hgb levels was fast and observed within a month or two after iv iron administration, and it was more than 1 g/dL. Only 18 out of 63 patients (28.5 %) needed blood transfusion due to further decrease in their Hgb level and the appearance of anemia symptoms within 3 months after iv iron administration. Iv iron prevented high blood transfusion rates in this patient population since a decrease in Hgb level, and a necessity of blood transfusion would be inevitable with the effect of cancer treatment probably in all of these patients in the course of time. Although the study was not randomized and not designed with a control group who did not receive iv iron, these patients have already generated their own controls with their Hgb levels before and after the administration of iv iron. We believe that without any intervention for anemia correction, the Hgb levels of most of these patients would gradually get worse with the effect of cancer treatment and disease per se.

Iv iron although popularized as an adjuvant to ESAs, is in fact nowadays the most evidence based alternative to both ESAs and blood transfusion in the treatment of cancer associated anemia. Acute life-threatening side effects and lethal anaphylactic reactions are the major concerns among clinicians with iv iron administration which is probably the most important factor limiting their usage [27, 28]. The most common difficulty encountered during our study was the fear of medical personnel in outpatient clinics to administer iv iron infusion to the patients. However according to United States Food and Drug Administration (FDA) on adverse drug events reports, life-threatening adverse drug events were 0.6 per million doses for iron sucrose, 0.9 for iron gluconate, and 3.3 for low molecular-weight iron dextran [25–28]. Life-threatening anaphylactic reactions as with older iron-dextran solutions have never been observed in cancer trials [5, 9]. Iron sucrose was reported to have the lowest adverse events especially the hypersensitivity reactions [29]. No serious adverse events have been observed in our patients during the study. A recent observational, prospective study performed in 367 patients with solid or hematologic tumors demonstrated the efficacy and safety of iv iron administration (ferric carboxymaltose) [30].

Another concern commonly present among clinicians is the fear of iron accumulation in patients with normal iron stores and elevated serum ferritin levels, However this fear is senseless due to the mechanism of functional iron deficiency, and due to the doses of iv iron administered in the treatment of cancer related anemia. Thus iv iron is still efficient in patients irrespective of serum iron and ferritin levels [9, 22]. In this study as well, patients responded to iv iron irrespective of their baseline serum iron and ferritin levels.

The deficiency in red blood cells and decreased functional capacity to deliver oxygen to tissues and low hemoglobin levels result in tumor hypoxia, conferring resistance to chemotherapy and radiotherapy, decreased local control, and ultimately decreased survival [31, 32]. Presence of anemia before cancer treatment and correction of anemia during cancer treatment is closely associated with survival [33]. We observed a statistically significant 1-year survival difference in metastatic patients with increased Hgb levels after iv iron administration during their cancer treatment with CT, RT, or both when compared to the patients without a response (61.1 % vs 35.3 %, *p* = 0.005). The survival advantage was also significant in metastatic patients who didn’t receive blood transfusion when compared to the ones who received blood transfusion (63 % vs 31.3 %, *p* = 0.004). However the survival figures should be evaluated with caution since the group was not homogenous with respect to treatment, patient and tumor characteristics. But increase in Hgb levels with iv iron administration may be both prognostic and predictive factor for survival in anemic cancer patients undergoing oncologic treatment. It is worth testing this hypothesis in a prospective trial.

The decrease in Hgb levels despite iv iron administration was observed less in patients with localized disease treated with adjuvant or curative intent in comparison to patients presenting with metastatic disease treated with palliative intent (3.7 vs 50 %, *p* < 0.001). We think that the lower response rate to iv iron in patients with metastatic disease is due to presence of higher tumor burden and associated presence of chronic inflammatory state and more release of inflammatory cytokines with respect to the patients with localized disease. High tumor burden and associated inflammation may increase the serum hepcidin levels in metastatic patients more than the hepcidin levels in patients with localized tumors. A recent study demonstrated that response to iv iron and erythropoietin is closely related to serum hepcidin levels [34].

We demonstrated a close relation between response to iv iron and response to cancer treatment. Tumor responses to cancer treatment in metastatic patients correlated with response to iv iron administration and this relation was statistically significant (*p* < 0.001). We hypothesize that iv iron response is predictive of response to oncologic treatment and it can predict response to oncologic treatment earlier than clinical and radiologic evaluation.

Increase in Hgb levels with iv iron administration were observed in all patients presenting with localized disease except one (1 out of 27 patients), and red blood cell transfusion was necessary only in this patient. Iv iron should be considered in all anemic cancer patients treated with adjuvant or curative intent since it is very effective and safe intervention with respect to blood transfusion. It is very important to prevent adverse effects of blood transfusion in curatively treated patients. We don’t know if iv iron administration provides a survival advantage in patients with localized disease as well, as in the metastatic patients, since all the patients except one had increased Hgb levels after iv iron and we need longer follow-up time in this group. Another important point to investigate in patients with localized tumors is the association of ulterior recurrences with the degree of Hgb increase, but we need more patients and longer follow-up time to demonstrate this interaction.

Anemia is a common problem in patients with cancer [3, 4, 35]. Although it has a negative impact on prognosis and treatment results, anemia is undertreated and is not a major concern among oncologists [4, 35]. The major reason behind this is the lack of effective treatment for anemia and the limitation of ESA’s usage with the understanding of their harms. However iv iron is a safe and effective treatment for anemia in patients even undergoing active cancer treatment either with CT, RT or both [30, 36]. Increase of hemoglobin with iv iron administration is cheap and safe, and it may prevent blood transfusion and its associated complications. Increase in Hgb levels by iv iron is not temporary as in blood transfusion, and may increase the survival in metastatic cancer patients receiving treatment for their cancer.

The major drawback in our retrospective study is the heterogeneity of the study population both in respect to the patient and treatment characteristics, but as a summary, anemia not responding to iv iron and necessitating further red blood cell transfusion indicates a worse prognosis and survival. It will be very promising and practice changing to show the same results in prospectively designed studies. However even in the absence of such studies, iv iron is a safe and best evidence based treatment alternative for anemic cancer patients especially during their oncologic treatment with CT, RT or both.

## Conclusions

Anemia is a common problem in cancer patients and it has negative impact on prognosis and treatment results. Transfusion of red blood cells is a common practice among oncologists for the treatment of cancer related anemia, and became popular again after the demonstration of harmful effects of erythropoiesis stimulating agents. However blood transfusion is not devoid of toxicity either. On the other hand intravenous administration of iron is a promising strategy and is reported to be effective for the treatment of anemia associated with malignancy. Amelioration of anemia with intravenous iron may result in increased response to cancer treatment and even better survival.

## Abbreviations

AID: Absolute iron deficiency
CRT: Concomitant chemoradiotherapy
CT: Chemotherapy
ESAs: Erythropoiesis-stimulating agents
FDA: Food and Drug Administration
FID: Functional iron deficiency
Hgb: Hemoglobin
IV: Intravenous
RECIST: Response evaluation criteria in solid tumors
RT: Radiotherapy
